# Detection and identification of putative bacterial endosymbionts and endogenous viruses in tick cell lines^☆^

**DOI:** 10.1016/j.ttbdis.2012.05.002

**Published:** 2012-06

**Authors:** M. Pilar Alberdi, Matthew J. Dalby, Julio Rodriguez-Andres, John K. Fazakerley, Alain Kohl, Lesley Bell-Sakyi

**Affiliations:** The Roslin Institute and Royal (Dick) School of Veterinary Studies, University of Edinburgh, Easter Bush, Roslin, Midlothian EH25 9RG, UK

**Keywords:** Tick cell line, Tick, Endosymbiont, Bacteria, Virus

## Abstract

As well as being vectors of many viral, bacterial, and protozoan pathogens of medical and veterinary importance, ticks harbour a variety of microorganisms which are not known to be pathogenic for vertebrate hosts. Continuous cell lines established from ixodid and argasid ticks could be infected with such endosymbiotic bacteria and endogenous viruses, but to date very few cell lines have been examined for their presence. DNA and RNA extracted from over 50 tick cell lines deposited in the Roslin Wellcome Trust Tick Cell Biobank (http://tickcells.roslin.ac.uk) were screened for presence of bacteria and RNA viruses, respectively. Sequencing of PCR products amplified using pan-16S rRNA primers revealed the presence of DNA sequences from bacterial endosymbionts in several cell lines derived from *Amblyomma* and *Dermacentor* spp. ticks. Identification to species level was attempted using *Rickettsia*- and *Francisella*-specific primers. Pan-Nairovirus primers amplified PCR products of uncertain specificity in cell lines derived from *Rhipicephalus*, *Hyalomma*, *Ixodes*, *Carios*, and *Ornithodoros* spp. ticks. Further characterisation attempted with primers specific for Crimean-Congo haemorrhagic fever virus segments confirmed the absence of this arbovirus in the cells. A set of pan-Flavivirus primers did not detect endogenous viruses in any of the cell lines. Transmission electron microscopy revealed the presence of endogenous reovirus-like viruses in many of the cell lines; only 4 of these lines gave positive results with primers specific for the tick Orbivirus St Croix River virus, indicating that there may be additional, as yet undescribed ‘tick-only’ viruses inhabiting tick cell lines.

## Introduction

Ixodid and argasid ticks are vectors of many viral, bacterial, and protozoan pathogens of worldwide medical and veterinary importance (Jongejan and Uilenberg, 2004). Some of these microorganisms are entirely dependent on ticks for transmission between vertebrate hosts in nature, such as tick-borne encephalitis virus (TBEV), *Borrelia* spp. spirochaetes, the obligatory intracellular genera *Rickettsia* and *Ehrlichia*, and the protozoan parasite genera *Babesia* and *Theileria*. Others, such as the obligatory intracellular bacterial genera *Coxiella*, *Francisella*, and some species of *Anaplasma*, can be naturally transmitted between vertebrates both by ticks and by other routes (direct contact, transplacental, biting flies) (Parola and Raoult, 2001; Aubry and Geale, 2011). A third group, including arboviruses such as Crimean-Congo haemorrhagic fever virus (CCHFV) and African swine fever virus (ASFV), are transmitted between wild vertebrates by ticks in a ‘sylvatic cycle’ without causing overt disease, but once they infect hosts such as humans or domestic pigs, respectively, direct vertebrate–vertebrate transmission can occur (Whitehouse, 2004; Costard et al., 2009). A fourth group of microorganisms cause persistent infections in ticks, presumably through transovarial transmission, but have no known vertebrate host. Many of these are only known through molecular detection by PCR and have not been visualised or cultivated in vitro, for example the *Francisella*-like endosymbionts detected in *Dermacentor*, *Amblyomma*, and *Ornithodoros* spp. ticks (Scoles, 2004).

Continuous cell lines derived from ixodid and argasid ticks are playing an increasingly important role in research on ticks and pathogenic and symbiotic tick-borne microorganisms (Bell-Sakyi et al., 2007, 2011). To date, very few of the around 60 currently available tick cell lines (Bell-Sakyi et al., 2011) have been screened for the presence of endosymbiotic bacteria and endogenous viruses. The *Dermacentor andersoni* embryo-derived cell line DAE100 was found to harbour the non-pathogenic endosymbiont *Rickettsia peacockii* (Simser et al., 2001), and a new species, *Rickettsia hoogstraalii*, was discovered in several cell lines derived from embryonic *Carios capensis* (Mattila et al., 2007). Najm et al. (2012) detected intermittent presence of DNA of the intramitochondrial symbiont *Candidatus* Midichloria mitochondrii in *Ixodes ricinus* and *Rhipicephalus* (*Boophilus*) *decoloratus* cell lines. A previously undescribed Orbivirus was found to infect the IDE2 cell line derived from embryonic *Ixodes scapularis* (Attoui et al., 2001); St Croix River virus (SCRV) is possibly the first ‘tick only’ virus to be discovered (Nuttall, 2009). Unidentified ‘reovirus-like particles’ were seen in electron micrographs of the *Rhipicephalus appendiculatus* nymph-derived cell line RA243 (Munz et al., 1987), but at the time there were no molecular tools available to assist in identification of this unknown, possibly endogenous virus. The effect of the presence of these endogenous microorganisms on growth of tick-borne pathogens in the infected cell lines is largely unknown, and similarly there is no information on their incidence in the remaining majority of tick cell lines.

Therefore, using PCR-based methods, we screened a representative panel of tick cell lines derived from embryonic, developing nymphal, or developing adult ixodid or argasid ticks for the presence of viruses and bacteria. DNA was extracted from the cells and screened with pan-bacterial 16S primers. RNA was used to generate cDNA which was screened using primers known to amplify tick-borne virus genera. PCR products were sequenced to aid identification. Selected cell lines were further examined by transmission electron microscopy (TEM) for presence of intracellular bacteria and viruses.

## Materials and methods

### Tick cell lines

Fifty continuous tick cell lines derived from species of the ixodid genera *Amblyomma*, *Dermacentor*, *Hyalomma*, *Ixodes*, and *Rhipicephalus*, and the argasid genera *Carios* and *Ornithodoros* (Table 1), were cultured in either 2.2 ml medium in flat-sided culture tubes (Nunc) or 5 ml medium in 25-cm^2^ flasks (Nunc), with weekly medium changes. The incubation temperatures used for each cell line are given in Table 1. For DNA extraction, cells were harvested either from actively growing cultures by pipetting followed by centrifugation at 200 × *g* for 5 min or from cryopreserved stabilates which were thawed rapidly, diluted 1 in 10 in appropriate complete medium and centrifuged as above. Cell pellets were resuspended in PBS. For RNA extraction, cells were harvested from actively growing cultures as described above; RNA extraction was not attempted from cryopreserved stabilates.

### DNA and RNA isolation

Total genomic DNA was prepared from harvested tick cells using the DNeasy Blood and Tissue Kit (Qiagen Ltd, Crawley, UK) following the manufacturer's protocol. Purified DNA was eluted from the spin column with 400 μl TE buffer (two successive 200-μl elutions) and stored at −20 °C until use. RNA was extracted using an RNeasy Midi Kit (Qiagen Ltd, Crawley, UK) following the manufacturer's instructions. Purified RNA was eluted using 300 μl (2 × 150 μl) RNase-free water stored at −80 °C until use.

### cDNA synthesis

First-strand cDNA synthesis was carried out using the SuperScript III System (Life Technologies, Inc.). Five hundred nanograms of total RNA and 1 μl of a 50-μM solution of random hexamers in a total volume of 13 μl were incubated for 10 min at 65 °C and chilled on ice. After adding 4 μl of 1st Strand Buffer, 1 μl of DTT (0.1 M), 2 μl of deoxynucleotide triphosphate mix (10 mM), and 1 μl of SuperScript reverse transcriptase III (200 units/μl), the reaction was incubated for 5 min at 25 °C, 1 h at 50 °C, and, finally, for 15 min at 70 °C. The resulting cDNA was used directly as a template for PCR amplification.

### PCR amplification

DNA and cDNA were amplified by PCR using primers listed in Table 2. Each 50 μl polymerase chain reaction (PCR) contained 36.85 μl molecular biology grade water (Sigma), 1 μl dNTP mix (10 mM of each dNTP), 10 μl 5 × PCR buffer, 0.2 μl of each primer (100 μM), 0.4 U of Promega GoTaq™ DNA polymerase, and 2 μl of template. Each PCR was carried out in an Applied Biosystems thermal cycler. Amplification was carried out with an initial 3-min denaturation at 95 °C followed by 40 cycles (55 cycles for the amplification of the Nairovirus S segment N ORF) of denaturation at 95 °C for 30 s, annealing at 50–60 °C for 30 s, and extension at 70 °C for 1 min 30 s. The amplification was completed by holding the reaction mixture for 7 min at 70 °C to allow complete extension. The PCR products were visualised by UV illumination on a 1% agarose gel stained with ethidium bromide.

### DNA sequencing and analysis

Positive PCR products of, or close to, the expected size were purified using a QIAquick PCR purification kit (Qiagen Ltd, Crawley, UK) following the manufacturer's recommendations. DNA sequencing in the forward and reverse directions was performed by DNA Sequencing & Services, MRCPPU, College of Life Sciences, University of Dundee, Scotland (www.dnaseq.co.uk) using Applied Biosystems Big-Dye Ver 3.1 chemistry on an Applied Biosystems model 3730 automated capillary DNA sequencer. Homology searches were performed in the NCBI database using the BLAST search programme (Altschul et al., 1990). Sequences were aligned using ClustalW software (Larkin et al., 2007) for multiple sequence alignment. Nucleotide sequence identities were calculated using Lasergene software (DNASTAR Inc., Madison, USA).

### Transmission electron microscopy

For TEM, samples of resuspended cells from selected cell lines were washed once in PBS, fixed in 3% glutaraldehyde in cacodylate buffer for 2–3 h, post-fixed in 1% osmium tetroxide in cacodylate buffer, dehydrated in acetone, and embedded in Araldite resin. Sections were cut on a Reichert OMU4 ultramicrotome (Leica), stained in uranyl acetate and lead citrate, and viewed in a Phillips CM120 transmission electron microscope. Images were taken on a Gatan Orius CCD camera.

## Results

### Molecular identification of putative bacterial endosymbionts in tick cell lines

When screened with pan-bacterial 16S rRNA primers, 5 of the tick cell lines from the genera *Amblyomma* and *Dermacentor* yielded PCR products in the expected size range of 528 bp (Table 2). BLAST analysis of the sequenced PCR products placed the putative bacteria in the genera *Rickettsia* (from the 2 *A. variegatum* cell lines) and *Francisella* (from 3 *Dermacentor* spp. cell lines). Further analysis with genus-specific primers (Table 2) yielded PCR products whose partial gene sequences were 98.2–99.8% identical to published sequences for, respectively, *Rickettsia africae* in AVL/CTVM13 and AVL/CTVM17, *Francisella*-like endosymbiont of *D. albipictus* in DALBE3 and *Francisella*-like endosymbiont of *D. variabilis* in DVE1 (Table 3). The sequence amplified from the *D. nitens* cell line ANE58 using pan-bacterial 16S rRNA primers showed nearly 97% similarity to sequences from endosymbionts of *O. moubata* and *D. variabilis* (Table 3). The sequences obtained from AVL/CTVM17 (16S rRNA, ompB and sca4 genes), ANE58 (16S rRNA), and DALBE3 (16S rRNA and lipoprotein genes) have been deposited in GenBank with accession numbers JX101606, JX101599, JX101598, JX101604, JX101605, and JX101603, respectively.

### Molecular detection of endogenous viruses in tick cell lines

**Fig. 1:**
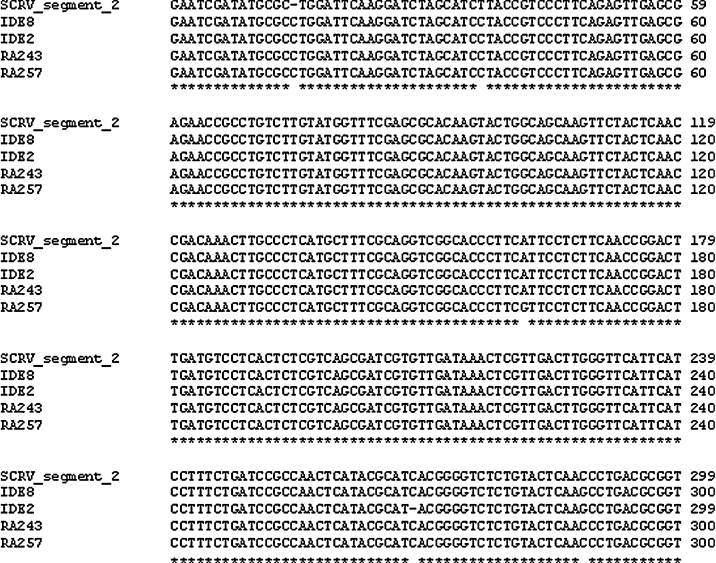
St Croix River virus segment 2 VP2 gene sequence alignment (ClustalW, using default parameters). The isolates from the 4 tick cell lines IDE2, IDE8, RA243, and RA257 show 98–99% similarity with the published sequence (GenBank accession number NC_005998).

As expected (Attoui et al., 2001; Bell-Sakyi et al., 2007), the *I. scapularis* cell lines IDE2 and IDE8 were PCR-positive for SCRV. The remaining 3 lines from this species, IDE12, ISE6, and ISE18 were negative for SCRV. Two *R. appendiculatus* cell lines, RA243 and RA257, were also positive in the SCRV PCR. All the SCRV PCR products were 98–99% identical to a 358-bp section of the SCRV segment 2 VP2 gene (Fig. 1). The sequences obtained from IDE8, RA243, and RA257 have been deposited in GenBank with accession numbers JX101600, JX101601, and JX101602, respectively.

**Fig. 2:**
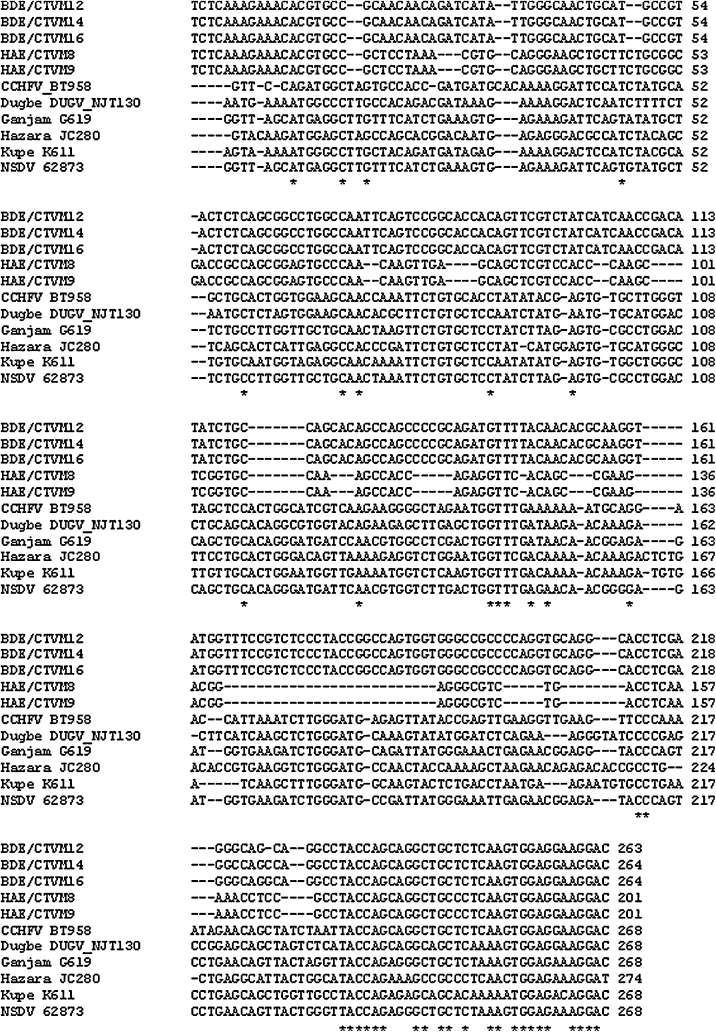
Alignment of sequences (ClustalW, using default parameters) from putative nairoviruses in tick cell lines BDE/CTVM12, 14 and 16 and HAE/CTVM8 and 9 with published sequences of the nairoviruses CCHF (CCHF BT958, EF123122), Dugbe (DUGV_NJT130, FJ422213), Ganjam (G619, AF504294), Hazara (JC280, M86624), Kupe (K611, EU257626), and Nairobi sheep disease (NSDV strain 62873, HQ286609).

Thirteen of the cell lines derived from the tick species *R.* (*B.*) *decoloratus* (BDE/CTVM12, 14, and 16), *R. appendiculatus* (RAE/CTVM1, RAN/CTVM3, and RA243), *H. anatolicum* (HAE/CTVM8 and 9), *I. scapularis* (IDE12), *C. capensis* (CCE1) and *O. moubata* (OME/CTVM21, 22, and 27) yielded PCR products of, or near the expected 400 bp size with the pan-Nairovirus primers from both of 2 cDNA samples generated on separate occasions. When sequenced, most were found to contain a short segment which was identical to nucleotides 431–463 of the nucleocapsid gene of the S genome segment of CCHFV. However, the remainder of each of the tick cell line-derived sequences did not match well with any other part of the genomes of CCHFV or other published nairoviruses, and it was not clear whether the amplification was specific or non-specific. In some cases, the sequence obtained with the forward primer differed from that obtained with the reverse primer. Considering the sequences obtained from both the forward and reverse primers, all the sequences were different from each other (Table 4) apart from the 3 *R.* (*B.*) *decoloratus* and the 2 *H. anatolicum* cell lines which yielded consensus sequences from both primers which were, respectively, 98% and 100% identical within tick species, but only 40% identical between species and with less than 38% identity to the published Nairovirus sequences (Fig. 2). PCR using CCHFV-specific primers did not amplify any specific products from cDNA generated from 10 of the cell lines, representative of all the tick species positive with the pan-Nairovirus primers (data not shown). The pan-Flavivirus primers did not reveal the presence of any endogenous viruses in the tick cell lines (data not shown).

### Detection of endogenous viruses in tick cell lines by electron microscopy

**Fig. 3:**
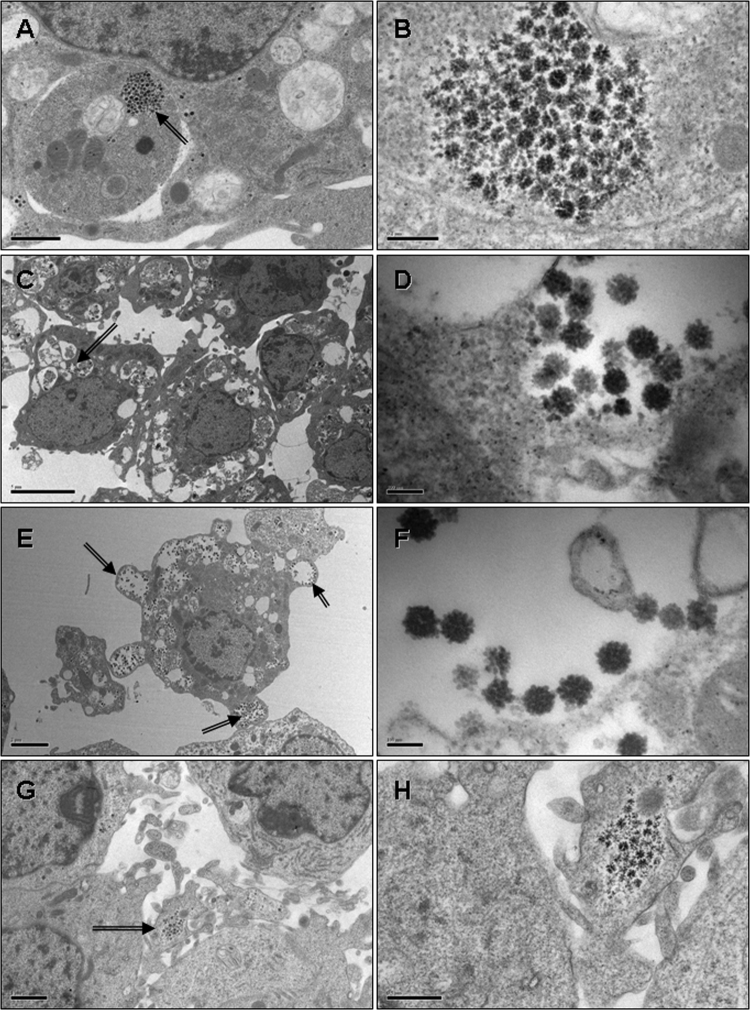
Transmission electron micrographs of 4 tick cell lines showing endogenous viruses (arrows). A (scale bar 1 μm) and B (scale bar 0.2 μm): *Amblyomma variegatum* cell line AVL/CTVM13; C (scale bar 5 μm) and D (scale bar 100 nm): *Rhipicephalus (Boophilus) microplus* cell line BDE/CTVM23; E (scale bar 2 μm) and F (scale bar 100 nm): *Dermacentor albipictus* cell line DALBE3; G (scale bar 1 μm) and H (scale bar 0.5 μm): *Dermacentor variabilis* cell line DVE1.

Samples of 35 of the ixodid tick cell lines were examined by TEM (Table 1), and 25 lines were found to harbour endogenous viruses which morphologically resembled reoviruses (Table 1, Fig. 3). No convincing bunyavirus-like particles were seen in any of the samples. The cell lines which were PCR-positive for *Rickettsia* (AVL/CTVM13 and AVL/CTVM17) or *Francisella*-like endosymbionts (DALBE3, DVE1, and ANE58) were included in this study, but no convincing evidence of intracellular bacteria was seen in any of the 5 lines.

## Discussion

Ticks have been found to harbour a wide range of endosymbiotic bacteria of genera including *Rickettsia* (Bell et al., 1963; Niebylski et al., 1997), *Francisella* (Scoles, 2004), *Midichloria* (Sassera et al., 2006), *Coxiella* (Noda et al., 1997), *Diplorickettsia* (Mediannikov et al., 2010), and *Arsenophonus* (Mediannikov et al., in press). Therefore, it is not surprising that several tick cell lines have previously been found to harbour apparently endosymbiotic *Rickettsia* spp. (Simser et al., 2001; Mattila et al., 2007). Indeed, one might have expected to find a higher proportion of the cell lines in the present study to be infected with bacteria than the 10% (5/50) that we detected using pan-bacterial primers. A possible explanation for this low prevalence could be provided by the finding of Alberdi et al. (in press) that apparently endosymbiotic *Rickettsia raoultii* eventually destroyed *Dermacentor reticulatus* embryo-derived primary cell cultures, preventing cell line initiation. If presence of intracellular endosymbionts in tick embryos used for primary culture initiation is a common occurrence, this may help to explain the low success rate historically experienced by tick tissue culturists (Varma, 1989; Bell-Sakyi et al., 2007).

Although we detected the presence of *Rickettsia* and *Francisella* spp. DNA in several tick cell lines, we were unable to detect bacteria in these cells by light (data not shown) or electron microscopy. We have attempted to infect *Amblyomma americanum* (AAE2) and *R.* (*B*.) *microplus* (BME/CTVM2) cells with the putative *Rickettsia* from the *A. variegatum* cell lines, also without success (data not shown). Therefore, we cannot say with certainty that these endosymbiotic bacteria are growing in the host cell lines. A similar problem arose in the study of Najm et al. (2012), who intermittently detected a small fragment of a *Candidatus* M. mitochondrii gene in 2 tick cell lines (BDE/CTVM14 and IRE/CTVM19), but failed to amplify a larger section of a different *Candidatus* M. mitochondrii gene from the same samples. Incorporation of segments of *Wolbachia* genes in invertebrate host genomes has been reported by McNulty et al. (2010) in nematodes and Martin et al. (2010) in woodlice; it is possible that a similar phenomenon occurs in ticks. On the other hand, endosymbiotic bacteria may be present in tick cell lines at such low levels as to be only intermittently detectable even by PCR, which is much more sensitive than microscopy.

Further studies, involving PCR targeting additional genes and more extensive subinoculation experiments, are required to definitively characterise and, if possible propagate in vitro, the putative endosymbiotic bacteria identified in the *Amblyomma* and *Dermacentor* spp. cell lines. The availability of systems for propagation of such endosymbionts in tick cell lines will aid in their characterisation, providing a useful model system for study of the interaction between the bacteria and their arthropod vectors. Understanding the mechanisms by which ticks and endosymbionts interact might reveal ways in which the non-pathogenic endosymbionts can be manipulated for control of both the ticks and the pathogens that they also harbour, as demonstrated for mosquito cell-adapted *Wolbachia pipientis* which, when transfected into *Aedes aegypti* mosquitoes, interfered with replication of the human pathogens Dengue virus, Chikungunya virus, and *Plasmodium* (Iturbe-Ormaetxe et al., 2011).

Nuttall (2009) lists 61 viruses transmitted by ticks – apart from the Orbivirus SCRV, these are viruses that have been detected because they infect vertebrate cells. To date, SCRV is the only characterised tick virus (Attoui et al., 2001); but it is likely that there are others (Nuttall, 2009). SCRV is known to chronically infect 2 *I. scapularis* cell lines, IDE2 (Attoui et al., 2001) and IDE8, both derived from progeny of field ticks collected from deer in Wisconsin, USA (Munderloh et al., 1994). Interestingly, we found that a third cell line, IDE12, which originated from the same area, was negative for SCRV, while 2 cell lines derived from the African species *R. appendiculatus* were positive in the PCR targeting segment 2 of this virus. The latter lines were both established in a laboratory in the UK by Varma et al. (1975) many years before the SCRV-positive *I. scapularis* lines were established by Munderloh et al. (1994) in a laboratory in the US; however, both RA243 and RA257 were subsequently cultivated in laboratories where IDE2 and/or IDE8 are also maintained, before deposition in the Tick Cell Biobank. On the other hand, reovirus-like particles were reported to be present in RA243 maintained in a laboratory in Germany by Munz et al. (1987), and we have successfully amplified a PCR product with 99% sequence identity to a 358-kb fragment of segment 2 of SCRV from a sample of RA243 cryopreserved since 1982 at Texas A&M University, where *I. scapularis* cell lines have never been cultivated (authors’ unpublished data; Patricia Holman, pers. communication). Further study is required to establish the origin and identity of the virus(es) in RA243 and RA257, and their relationship to the SCRV in IDE2 and IDE8.

We found reovirus-like particles in all of the *I. scapularis* cell lines examined except IDE12, but did not observe any in the RA243 cells, suggesting that if SCRV is replicating in this cell line, it is present at a low level compared to the viruses in the *I. scapularis* cells, and/or it only infects a small proportion of the total cell population. We cannot definitely say that the reovirus-like particles observed in IDE2 and IDE8 cells are in fact SCRV, and the viruses in ISE6, ISE18, and the 21 other cell lines in which we saw reovirus-like particles remain at present unidentified. We could not find any reports of TEM detection of reoviruses or reovirus-like particles in tick cell lines, apart that from Munz et al. (1987). However, structures described as ‘deposits of beta particles of glycogen’ in salivary glands of partially-fed *R. appendiculatus* ticks infected with *Theileria parva* by Fawcett et al. (1982) closely resemble some of our putative endogenous viruses. Other TEM studies report presence of glycogen in perineurial cells of engorged female *R.* (*B.*) *microplus* (Binnington and Lane, 1980) and midgut cells of unfed adult *A. americanum* (Jaworski et al., 1983) but, in these reports, the magnification of the electron micrographs is insufficient to detect any structural detail within the glycogen particles. Although beta particles of glycogen as visualised by TEM appear to be of a similar texture to some of our putative viruses (particularly those shown in Fig. 2b, d, and f), they resemble random agglomerations of glycogen alpha particles, occur in a wide range of sizes, and are highly pleomorphic whilst retaining similar electron density throughout, as illustrated previously (Rhodin, 1974). In contrast, our putative viruses are generally roughly spherical, radially or at least bilaterally symmetrical with regular arrangements of external protrusions, and are of a similar size and electron density when in the same plane of section within each group of particles. A possible approach to resolving the identity of these virus-like particles would be to apply differential fixation protocols to render glycogen particles invisible in TEM preparations as described by Binnington and Lane (1980).

Many of the known tick-borne viruses belong to the families Bunyaviridae (genus Nairovirus), Flaviviridae (genus Flavivirus), and Reoviridae (genus Orbivirus). Therefore we carried out a preliminary screening of the tick cell lines using one set each of pan-Nairovirus and pan-Flavivirus primers. Due to time restrictions, we were unable to include additional primer sets such as those of Palacios et al. (2011) targeting orbiviruses. The high incidence of reovirus-like viruses detected by TEM suggests that these pan-Orbivirus primers are likely to aid us in detection and identification of endogenous viruses in many of the tick cell lines.

Nuttall (2009) lists 19 tick-borne bunyaviruses, of which most either belong in the Nairovirus and Phlebovirus genera, or are unassigned. Of the 7 primer pairs designed by Lambert and Lanciotti (2009) for detection of different Bunyavirus groups, we only tested the Nairovirus primers. In future, their primer pairs targeting phleboviruses and orthobunyaviruses, of which at least one member is tick-borne, should also be used to screen the tick cell lines. Using the Nairovirus primers, we amplified PCR products from 13 cell lines derived from 3 ixodid and 2 argasid tick genera, but due to the high degree of diversity and low level of sequence identity with the few published Nairovirus sequences, it was difficult to conclude anything from the sequences of many of them, except that they indicated the possible presence of one or more novel nairoviruses in each of the cell lines. On 2 occasions, an almost identical sequence was amplified from the 3 *R.* (*B.*) *decoloratus* cell lines, which were all derived from the same pool of 4 egg batches, suggesting that the same virus may be present in all 3 lines. Similarly, almost identical sequences were amplified from the 2 *H. anatolicum* lines suggesting that they may also harbour the same virus, although they were derived from different egg batches (Bell-Sakyi, 1991). In contrast, sequences amplified using the forward and reverse primers differed for the 3 *O. moubata* lines and 2 of the *R. appendiculatus* lines (RAE/CTVM1 and RAN/CTVM3), suggesting that these lines could each harbour more than one virus. The negative results obtained using the CCHFV-specific primer sets confirmed the absence of this particular Nairovirus in the tick cell lines. Further analysis is needed to identify and characterise these new putative nairoviruses and to determine whether they are arboviruses with the potential to infect vertebrate hosts, or tick-only viruses. The primers targeting the L polymerase-encoding region of the Nairovirus genome, used by Honig et al. (2004) to amplify and sequence PCR products from 14 different tick-borne nairoviruses, could assist us in this future analysis.

The pan-Flavivirus primers which we used are reported to consistently amplify several flaviviruses (Johnson et al., 2010); however these authors only included 2 closely-related tick-borne viruses, TBEV and louping ill virus, in their test panel, so it is possible that this assay might not detect endogenous flaviviruses harboured by the tick cell lines. Screening with additional broad-spectrum primers amplifying different portions of the Flavivirus genome and proven to detect a wide range of tick-borne flaviviruses (Moureau et al., 2008; Maher-Sturgess et al., 2008), should be carried out in future. Ngoye virus, a novel Flavivirus, was detected in *Rhipicephalus* spp. ticks using such a PCR-based method (Grard et al., 2006). This virus failed to replicate in cell lines from vertebrate and tick species, including a non-host *Rhipicephalus* sp., suggesting the possibility that it might be another ‘tick virus’. Moreover, the recent detection of ASFV-like gene sequences in human serum (Loh et al., 2009) suggests that the tick-borne DNA virus ASFV may not be the only member of the family Asfaviridae and that screening DNA from the tick cell lines for ASFV-related sequences might also be productive.

Over half of the known tick-borne viruses have been propagated in tick cells, along with many insect-borne arboviruses (Bell-Sakyi et al., 2011). Infection of tick cell lines with arboviruses generally results in low-level persistent infection, in the absence of detectable cytopathic effects. The cellular and molecular mechanisms responsible for this tolerance of arbovirus infection by tick cells are unknown; it is possible that ticks have evolved to coexist with a community of endogenous viruses. The continuous presence of these tick viruses may moderate or suppress the tick cell innate immune response to arbovirus infection, allowing persistent infection to occur. Ongoing studies in our laboratories to dissect the tick cell innate immune response to arbovirus infection (Bell-Sakyi et al., 2011) will help to elucidate the role of endogenous viruses in these processes.

## Figures and Tables

**Table 1:** Continuous tick cell lines examined in this study. All the lines are deposited in the Tick Cell Biobank (http://tickcells.roslin.ac.uk).

Tick species	Cell line	Instar	Incubation temperature	Reference	Cell samples examined		
					DNA	RNA	TEM
*Amblyomma americanum*	AAE2	Embryo	32 °C	Kurtti et al. (2005)	+	+	+^a^
	AAE12	Embryo	32 °C	Singu et al. (2006)	+	+	+^a^
*A. variegatum*	AVL/CTVM13	Developing nymph	32 °C	Bell-Sakyi et al. (2000)	+	+	+^a^
	AVL/CTVM17	Developing nymph	32 °C	Bell-Sakyi (2004)	+	+	+^a^
*Rhipicephalus (Boophilus) decoloratus*	BDE/CTVM12	Embryo	32 °C	Lallinger et al. (2010)	+	+	ND
	BDE/CTVM14	Embryo	28 °C	Lallinger et al. (2010)	+	+	+^a^
	BDE/CTVM16	Embryo	28 °C	Bell-Sakyi (2004)	+	+	+
*R. (Boophilus) microplus*	BME/CTVM2	Embryo	28 °C	Bell-Sakyi (2004)	+	+	+
	BME/CTVM4	Embryo	28 °C	Bell-Sakyi et al. (2007)	+	+	ND
	BME/CTVM5	Embryo	28 °C	Bell-Sakyi et al. (2007)	+	+	+^a^
	BME/CTVM6	Embryo	28 °C	Bell-Sakyi (2004)	+	+	+
	BME/CTVM23	Embryo	32 °C	Alberdi et al. (in press)	+	+	+^a^
	BME/CTVM30	Embryo	28 °C	Bell-Sakyi (unpublished)	+	+	+^a^
	BmVIII-SCC	Embryo	32 °C	Holman (1981)	+	+	+
	BME26	Embryo	32 °C	Kurtti et al. (1988)	ND	+	ND
*R. appendiculatus*	RAE/CTVM1	Embryo	32 °C	Bell-Sakyi (2004)	+	+	+^a^
	RAN/CTVM3	Developing adult	28 °C	Bekker et al. (2002)	+	+	+^a^
	RA243	Developing adult	32 °C	Varma et al. (1975)	+	+	+
	RA257	Developing adult	28 °C	Varma et al. (1975)	+	+	ND
*R. evertsi*	REE/CTVM28	Embryo	28 °C	Bell-Sakyi (unpublished)	+	+	+^a^
	REE/CTVM29	Embryo	28 °C	Bell-Sakyi (unpublished)	+	+	+^a^
	REE/CTVM31	Embryo	28 °C	Bell-Sakyi (unpublished)	+	+	+^a^
*R. sanguineus*	RSE8	Embryo	32 °C	Kurtti et al. (1982)	+	+	+^a^
*Dermacentor andersoni*	DAE15	Embryo	32 °C	Kurtti et al. (2005)	+	+	+
	DAE100T	Embryo	32 °C	Simser et al. (2001)	+	+	+
*D. albipictus*	DALBE3	Embryo	32 °C	Kurtti et al. (2005)	+	+	+^a^
*D. nitens*	ANE58	Embryo	32 °C	Kurtti et al. (1983)	+	+	+^a^
*D. variabilis*	DVE1	Embryo	32 °C	Kurtti et al. (2005)	+	+	+^a^
	RML-15	Embryo	28 °C	Yunker et al. (1981)	+	+	+
*Hyalomma anatolicum*	HAE/CTVM7	Embryo	ND	Bell-Sakyi (1991)	+	ND	ND
	HAE/CTVM8	Embryo	32 °C	Bell-Sakyi (1991)	+	+	+
	HAE/CTVM9	Embryo	32 °C	Bell-Sakyi (1991)	+	+	+^a^
	HAE/CTVM10	Embryo	ND	Bell-Sakyi (1991)	+	ND	ND
	HAE/CTVM11	Embryo	ND	Bell-Sakyi (1991)	+	ND	ND
*Ixodes ricinus*	IRE/CTVM19	Embryo	28 °C	Bell-Sakyi et al. (2007)	+	+	+^a^
	IRE/CTVM20	Embryo	28 °C	Bell-Sakyi et al. (2007)	+	+	+^a^
	IRE11	Embryo	32 °C	Simser et al. (2002)	+	+	+^a^
*I. scapularis*	IDE2	Embryo	32 °C	Munderloh et al. (1994)	+	+	+^a^
	IDE8	Embryo	32 °C	Munderloh et al. (1994)	+	+	+^a^
	IDE12	Embryo	32 °C	Munderloh et al. (1994)	+	+	+
	ISE6	Embryo	32 °C	Kurtti et al. (1996)	+	+	+^a^
	ISE18	Embryo	32 °C	Munderloh et al. (1994)	+	+	+^a^
*Ornithodoros moubata*	OME/CTVM21	Embryo	28 °C	Bell-Sakyi et al. (2009)	+	+	ND
	OME/CTVM22	Embryo	28 °C	Bell-Sakyi et al. (2009)	+	+	ND
	OME/CTVM24	Embryo	28 °C	Bell-Sakyi et al. (2009)	+	+	ND
	OME/CTVM25	Embryo	28 °C	Bell-Sakyi et al. (2009)	+	+	ND
	OME/CTVM26	Embryo	28 °C	Bell-Sakyi et al. (2009)	+	+	ND
	OME/CTVM27	Embryo	28 °C	Bell-Sakyi et al. (2009)	+	+	ND
*Carios capensis*	CCE1	Embryo	32 °C	Mattila et al. (2007)	+	+	ND
	CCE2	Embryo	32 °C	Mattila et al. (2007)	+	+	ND

ND, not done.

a Reovirus-like particles seen.

**Table 2:** Oligonucleotide primers used in this study for the amplification and sequencing of PCR products.

Target/location	Forward primer	Reverse primer	Amplicon	Reference
Pan-bacterial/16S rRNA	AGAGTTTGATCCTGGCTCAG	GWATTACCGCGGCTGCTGG	528 bp	Benson et al. (2004)
*Rickettsia*/16S rRNA	GAACGCTATCGGTATGCTTAACACA	CATCACTCACTCGGTATTGCTGGA	364 bp	Nijhof et al. (2007)
*Rickettsia*/*ompB*	AAACAATAATCAAGGTACTGT	TACTTCCGGTTACAGCAAAGT	790 bp	Roux and Raoult (2000)
*Rickettsia*/*sca4*	ATGAGTAAAGACGGTAACCT	AAGCTATTGCGTCATCTCCG	900 bp	Sekeyova et al. (2001)
*Francisella*/lipoprotein	GAATATGTCAAAGGTAGG	TCAGAAGCGATTACTTCT	838 bp	Sjöstedt et al. (1990)
SCRV/segment 2	CGCATCAAGGGTGGGGGCTG	CAAGCAACCCAGGAGGGCGG	358 bp	Attoui et al. (2001)
Pan-*Flavivirus*/NS5	GCMATHTGGTWCATGTGG	GTRTCCCAKCCDGCNGTRTC	203 bp	Johnson et al. (2010)
*Nairovirus*/S segment N ORF	TCTCAAAGAAACACGTGCCGC	GTCCTTCCTCCACTTGWGRGCAGCCTGCTGGTA	400 bp	Lambert and Lanciotti (2009)
CCHFV/S segment	TCTCAAAGAAACACGTGCCGC	TCTCAAAGATATCGTTGCCGC	1.6 kb	Deyde et al. (2006)
CCHFV/M segment	TCTCAAAGAAATACTTGC	TCTCAAAGATATAGTGGC	5.4 kb	Deyde et al. (2006)
CCHFV/L segment (L1)	TCTCAAAGATATCAATCCCCCC	TTGGCACTATCTTTCATTTGAC	6 kb	Deyde et al. (2006)
CCHFV/L segment (L2)	GAAGAGCTATATGACATAAGGC	TCTCAAAGAAATCGTTCCCCCCAC	6 kb	Deyde et al. (2006)

**Table 3:** Identification of bacterial sequences detected by PCR in cell lines derived from *Amblyomma variegatum* (AVL/CTVM13 and AVL/CTVM17), *Dermacentor albipictus* (DALBE3), *D. nitens* (ANE58), and *D. variabilis* (DVE1).

Cell line	Primer set	Top three matches with GenBank sequences (% identity, accession number)		
		1	2	3
AVL/CTVM13	Pan-bacterial 16S rRNA	*Rickettsia africae* clone 1.2 (99.5, JF949789)	*Rickettsia africae* clone 4.1 (98.8, JF949792)	*Rickettsia africae* clone 2.1x (98.6, JF949790)
	*Rickettsia* 16S rRNA	*Rickettsia africae* clone 1.2 (99.7, JF949789)	*Rickettsia africae* clone 4.1 (99.3, JF949792)	*Rickettsia africae* clone 2.1x (99.0, JF949790)
	*Rickettsia ompB*	*Rickettsia africae* ESF-5 (99.0, CP001612)	*Rickettsia africae* OmpB (ompB) (99.0, AF123706)	*Rickettsia parkeri* OmpB (ompB) (98.4, AF123717)
	*Rickettsia sca4*	*Rickettsia africae* ESF-5 (98.4, CP001612)	*Rickettsia africae* cell surface antigen (sca4) gene (98.4, AF151724)	*Rickettsia honei* subsp. *marmionii* PS 120 antigen gene (97.6, DQ309095)
AVL/CTVM17	Pan-bacterial 16S rRNA	*Rickettsia africae* clone 1.2 (99.6, JF949789)	*Rickettsia africae* clone 4.1 (98.9, JF949792)	*Rickettsia africae* clone 2.1x (98.8, JF949790)
	*Rickettsia* 16S rRNA	*Rickettsia africae* clone 1.2 (99.8, JF949789)	*Rickettsia africae* clone 4.1 (99.7, JF949792)	*Rickettsia africae* clone 2.1x (99.0, JF949790)
	*Rickettsia ompB*	*Rickettsia africae* ESF-5 (98.8, CP001612)	*Rickettsia africae* OmpB (ompB) (98.8, AF123706)	*Rickettsia parkeri* OmpB (ompB) (98.2, AF123717)
	*Rickettsia sca4*	*Rickettsia africae* ESF-5 (98.3, CP001612)	*Rickettsia africae* cell surface antigen (sca4) gene (98.3, AF151724)	*Rickettsia honei* subsp. *marmionii* PS 120 antigen gene (97.5, DQ309095)
DALBE3	Pan-bacterial 16S rRNA	*Francisella* endosymbiont of *Dermacentor albipictus* clone T1G.E16s 16S ribosomal RNA gene (99.6, AY375394)	*Francisella* endosymbiont of *Dermacentor andersoni* clone 01-171.E16s 16S ribosomal RNA gene (99.4, AY375397)	*Ornithodoros moubata* symbiote B gene for 16S rRNA (99.0, AB001522)
	*Francisella* lipoprotein	*Francisella*-like endosymbiont of *Dermacentor albipictus* haplotype 5 17 kDa lipoprotein gene (98.2, GU968877)	*Francisella*-like endosymbiont of *Dermacentor albipictus* haplotype 3 17 kDa lipoprotein gene (98.2, GU968875)	*Francisella* endosymbiont of *Dermacentor albipictus* clone 02-045 17 kDa lipoprotein gene (98.2, AY375409)
ANE58	Pan-bacterial 16S rRNA	*Ornithodoros moubata* symbiote B gene for 16S rRNA (98.8, AB001522)	*Francisella* cf. *novicida* Fx1 (96.6, CP002557)	*Francisella* cf. *novicida* 3523 (96.6, CP002558)
	*Francisella* lipoprotein	Negative by PCR		
DVE1	Pan-bacterial 16S rRNA	*Francisella* sp. DVFSQ83_04 16S ribosomal RNA gene from *D. variabilis* (99.2, AY795977)	*Francisella* sp. DVFSQ81_04 16S ribosomal RNA gene from *D. variabilis* (98.9, AY795976)	*Francisella* sp. DVFSQ80_04 16S ribosomal RNA gene from *D. variabilis* (98.9, AY795978)
	*Francisella* lipoprotein	Negative by PCR		

**Table 4:**
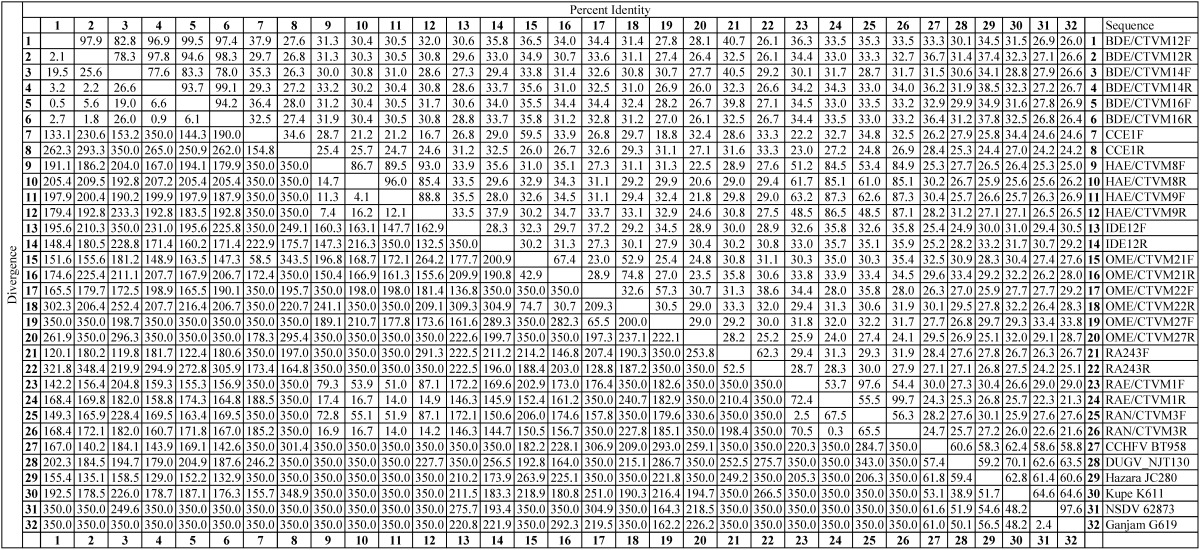
Nucleotide sequence identity differences of the putative tick cell nairoviruses amplified from 13 tick cell lines using pan-Nairovirus forward (F) and reverse (R) primers and published sequences of the nairoviruses CCHF (CCHF BT958, EF123122), Dugbe (DUGV_NJT130, FJ422213), Hazara (JC280, M86624), Kupe (K611, EU257626), Nairobi sheep disease (NSDV strain 62873, HQ286609), and Ganjam (G619, AF504294).
